# Salidroside Exerts Beneficial Effect on Testicular Ischemia-Reperfusion Injury in Rats

**DOI:** 10.1155/2022/8069152

**Published:** 2022-05-11

**Authors:** Si-Ming Wei, Yu-Min Huang, Zhi-Quan Qin

**Affiliations:** ^1^Shulan International Medical College, Zhejiang Shuren University, Hangzhou, Zhejiang, China 310015; ^2^School of Nursing, Zhejiang Chinese Medical University, Hangzhou, Zhejiang, China 310053; ^3^Department of Sport Science, College of Education, Zhejiang University, Hangzhou, Zhejiang, China 310058; ^4^Cancer Center, Department of Medical Oncology, Zhejiang Provincial People's Hospital (Affiliated People's Hospital, Hangzhou Medical College), Hangzhou, Zhejiang, China 310014

## Abstract

Testicular torsion-detorsion results in testicular ischemia-reperfusion injury, which is associated with overgeneration of reactive oxygen species. Salidroside, a major bioactive ingredient extracted from *Rhodiola rosea*, has strong antioxidant activity. The purpose of this study was to examine the effect of salidroside on testicular ischemia-reperfusion injury. Sixty rats were randomly separated into 3 experimental groups: group A = sham-operated control; group B = testicular ischemia-reperfusion; and group C = testicular ischemia-reperfusion treated with salidroside. The rats in the sham-operated control group received all surgical procedures except testicular torsion-detorsion. The testicular ischemia-reperfusion group underwent 2 hours of left testicular torsion followed by detorsion. The rats in the salidroside-treated group received the same surgical procedure as in testicular ischemia-reperfusion group, but salidroside was injected intraperitoneally at reperfusion. Testicular malondialdehyde content (a reliable index of reactive oxygen species) and protein expression of superoxide dismutase and catalase which are primary antioxidant enzymes in testes were measured at 4 hours after reperfusion. Testicular spermatogenesis was evaluated at 3 months after reperfusion. The malondialdehyde content increased significantly, while superoxide dismutase and catalase protein expression and testicular spermatogenesis reduced significantly in ipsilateral testes of testicular ischemia-reperfusion group, as compared with sham-operated control group. Therapy with salidroside significantly reduced malondialdehyde content and significantly enhanced superoxide dismutase and catalase protein expression and spermatogenesis in ipsilateral testes, as compared with testicular ischemia-reperfusion group. The present findings indicate that treatment with salidroside ameliorates testicular ischemia-reperfusion injury by reducing reactive oxygen species level by upregulating superoxide dismutase and catalase protein expression.

## 1. Introduction

As a common urological emergency, testicular torsion commonly affects around 1 in 4000 males aged <25 years [1]. Testicular torsion can occur at any age but usually occurs in young males, with a bimodal peak of incidence: during the first year of life and between the ages of 13 and 16 years [2]. It is caused by a twisting of the testis around the longitudinal axis of the spermatic cord, initially resulting in venous occlusion followed by arterial obstruction. A delay in diagnosis and treatment can lead to testicular infarction. Recent study has shown that if testicular torsion is not treated in time, rate of orchiectomies varies between 25% and 40% [3]. Early surgical detorsion is an optional maneuver to allow reperfusion of blood flow. Some studies have reported that 13.5%-68% of patients with timely detorsion eventually develop testicular atrophy [4–8]. Testicular damage after torsion-detorsion is thought to be a typical ischemia-reperfusion injury. Tissular ischemia-reperfusion causes production of large quantities of reactive oxygen species [9–12]. Reactive oxygen species, including superoxide anion, nitric oxide, peroxynitrite, hydrogen peroxide, and hydroxyl radical, can result in tissue damage by inducing lipid peroxidation in the cellular and mitochondrial membranes, DNA destruction, and protein denaturation [13]. As testicular tissue contains a large number of polyunsaturated fatty acids, it is highly susceptible to the detrimental effects of reactive oxygen species, particularly to lipid peroxidation [14, 15].

Currently, there is not an effective pharmacological agent to treat testicular ischemia-reperfusion injury in clinical practice. *Rhodiola rosea*, also called Arctic root or golden root, is a medicinal plant that grows in the Arctic and mountainous regions at high altitude in Asia, Europe, and America [16]. In China, *Rhodiola rosea* has been widely used as a traditional Tibetan medicine for thousands of years to promote blood circulation and treat angina, apoplexy, and asthma [17]. Salidroside is a major bioactive ingredient extracted from *Rhodiola rosea* [18]. Its molecular formula and molecular weight are C_14_H_20_O_7_ and 300.3, respectively [19]. Salidroside is documented to have a wide range of pharmacological effects, such as antioxidative, anti-inflammatory, antihypoxic, antifatigue, antidepressive, antiaging, and antitumor properties [20–26]. Various studies have confirmed that salidroside possesses ameliorative effect on ischemia-reperfusion injury in the heart, brain, liver, and spinal cord [27–30]. However, studies investigating the effect of salidroside on testicular ischemia-reperfusion injury are entirely absent. Therefore, the aim of the present study was to assess whether salidroside has a beneficial effect on ischemia-reperfusion injury in rat testis.

## 2. Materials and Methods

### 2.1. Animals and Ethics

Sixty male Sprague-Dawley rats weighing 250-300 g were supplied by Shanghai SLAC Laboratory Animal Co., Ltd. (Shanghai City, China). The animals were kept in plastic cages with sawdust bedding, which were placed in well-ventilated standard conditions (21°C ± 1°C, and 12-hour periods of light-dark exposure). Rats fed on standard rat chow and sterile water. The experimental protocols in this study were approved by the Animal Research Ethical Committee at our university (Approval No. 10790; approval date: March 18, 2019). All animal experiments were performed in accordance with the US National Institutes of Health guide for the care and use of laboratory animals.

### 2.2. Drugs and Chemicals

Salidroside, ketamine, primary antibody against *β*-actin, and hematoxylin and eosin were supplied by Sigma Chemical Company (St. Louis, MO, USA). A malondialdehyde analyzing kit was obtained from Nanjing Jiancheng Institute of Bioengineering (Nanjing City, China). Protein quantification kit was purchased from Bio-Rad Laboratories (Hercules, CA, USA). The primary antibodies against copper- and zinc-containing (Cu-Zn) superoxide dismutase and catalase, horseradish peroxidase-labelled secondary antibody, and an enhanced chemiluminescence kit were provided by Santa Cruz Biotechnology (Santa Cruz, CA, USA). The other chemicals used in the course of study were high-quality and commercially obtainable.

### 2.3. Testicular Ischemia-Reperfusion and Treatment

Sixty rats were randomly divided into 3 experimental groups (*n* = 20 per group): group A = sham-operated control; group B = testicular ischemia-reperfusion; and group C = testicular ischemia-reperfusion treated with salidroside. The surgical procedure was conducted in accordance with our previously described method [31]. Operation was carried out with ketamine (50 mg/kg, intraperitoneal) anesthesia under aseptic conditions. The scrotum was entered through a left-sided ilioinguinal incision, and the left testis was extracted gently. An 11-0 atraumatic silk suture was placed through tunica albuginea in the sham-operated control group. Then, the testis was positioned back into the scrotum, and the incision was closed in a single plane with a 4-0 silk suture. Rats in the testicular ischemia-reperfusion group had their left testes twisted at 720° in a counterclockwise direction so that testes were in a state of ischemia. The ischemia was maintained by fixing the testes medially and laterally to scrotal wall with 11-0 silk suture through the tunica albuginea. Two hours later, the twisted testes were released and restored to the natural position to induce reperfusion. The testes were still living for recirculation of blood flow and placed back into the scrotum. The rats in the salidroside-treated group received the same surgical procedure as in the testicular ischemia-reperfusion group, but salidroside (20 mg/kg) was intraperitoneally administered at reperfusion. The rationale for the dose of salidroside used in this study was based on previous reports [27–29]. As mentioned above, a total of 60 rats were randomly separated into three groups, each containing 20 rats. In each group, 10 rats underwent bilateral orchiectomy 4 hours after reperfusion for estimation of malondialdehyde content and superoxide dismutase and catalase protein expression; the other 10 rats received bilateral orchiectomy 3 months after reperfusion for determination of testicular spermatogenesis. All these rats were eventually euthanized by using special carbon dioxide device.

### 2.4. Malondialdehyde Analysis

Testicular tissue was homogenized in malondialdehyde lysis buffer and centrifuged at 5,000 × gravity for 15 minutes at 4°C. Then, the obtained supernatant was used to detect malondialdehyde content using a commercial kit according to instructions of the manufacturer. Malondialdehyde content was evaluated by measuring the thiobarbituric acid reactive substance as described by Ohkawa et al. [32]. The measurement method for malondialdehyde is based on the reaction of malondialdehyde with thiobarbituric acid to produce a pink-coloured chromogen. The absorption maximum peak of the sample was determined at 532 nm using a spectrophotometer. Malondialdehyde content is expressed as nmol/mg protein.

### 2.5. Western Blot Analysis for Superoxide Dismutase and Catalase Protein Expression

Testicular tissue sample was homogenized on ice in tissue protein extraction reagents containing 50 mM Tris HCl, pH 7.4, 0.5 *μ*g/ml leupeptin, 1% nonidet P-40, 5 *μ*g/ml aprotinin, 2 mM sodium orthovanadate, 1 mM dithiothreitol, 1 mM phenylmethylsulfonyl fluoride, 150 mM NaCl, 0.5% sodium deoxycholate, 0.1% sodium dodecyl sulfate, and 0.5 mM ethylenediaminetetraacetic acid. After incubation on ice for 30 minutes, the crud homogenate was centrifuged at 4°C for 15 minutes at 14,000 × gravity. The supernatant was harvested and used for assessment of protein concentration by using the Bradford protein assay kit [33]. Protein extract was denatured in boiling water for 3 minutes. Protein sample (20 *μ*g) was separated by sodium dodecyl sulfate polyacrylamide gel electrophoresis. The separated proteins were electrotransferred onto a nitrocellulose membrane. The unspecific binding sites on the membrane were blocked for 1 hour at room temperature with Tris-buffered saline containing 0.1% Tween-20 and 5% fat-free milk powder. Then, the membrane was incubated at 4°C overnight with primary antibody solution of Cu-Zn superoxide dismutase, catalase, or *β*-actin as an internal control. After the membrane was washed with Tris-buffered saline containing 0.1% Tween-20, it was incubated with secondary antibody coupled with horseradish peroxidase for 1 hour at room temperature. The membrane was washed again with Tris-buffered saline containing 0.1% Tween-20, and protein bands on the membrane were visualized using the enhanced chemiluminescence system followed by autoradiography. The optical intensity of Cu-Zn superoxide dismutase, catalase, and *β*-actin protein bands was measured by a GS-700 imaging densitometer (Bio-Rad Laboratories). The intensity ratio of Cu-Zn superoxide dismutase or catalase band to internal standard *β*-actin band from the same sample showed a relative expression level of Cu-Zn superoxide dismutase or catalase protein.

### 2.6. Measurement of Testicular Spermatogenesis

Testicular spermatogenesis was analyzed by measuring testicular weight, seminiferous tubular diameter, germ cell layer number, and Johnsen's testicular biopsy score [34]. The testes of rats in all three groups were removed, weighed, and placed in Bouin's fixative for four hours. Then, testicular tissue was dehydrated in an increasing concentration alcohol series and embedded in paraffin block. A 5 *μ*m thick section was cut from paraffin block by the use of a microtome and mounted on glass slide. After deparaffinization and staining with hematoxylin and eosin, the section was analyzed under an optical microscope at 200× magnification by the same pathologist blinded to the identity of each group. The seminiferous tubular diameter, germ cell layer number, and Johnsen's testicular biopsy score were assessed in 20 roundest seminiferous tubules from each testicular section. A light microscope-adaptable micrometer was used to determine seminiferous tubular diameter. We measured germ cell layer number in each seminiferous tubule by counting the number of germ cell layers from basal membrane to lumen of tubule at 90°, 180°, 270°, and 360°, and average number was calculated. The Johnsen's testicular biopsy score was used to evaluate maturity of germinal epithelium in the seminiferous tubule [35]. A score from one to ten was assigned to each tubule according to this system of evaluation. One point expresses no seminiferous epithelial cells. Ten points express full spermatogenesis with many spermatozoa, germinal epithelium of a regular thickness, and an open tubular lumen.

### 2.7. Data Analysis

Data were presented as mean ± standard deviation. We performed data analysis using GraphPad Prism software (Version 4.0; GraphPad Software Inc., San Diego, CA, USA). Normal distribution of data was confirmed by Shapiro-Wilk test. Data comparisons among groups were performed by using one-way analysis of variance with Student-Newman-Keuls multiple comparison test. The Student *t*-test was used to compare the results between ipsilateral and contralateral testes within group. Statistical significance was defined as *P* value of less than 0.05.

## 3. Results

### 3.1. Testicular Malondialdehyde Findings

**Figure 1:**
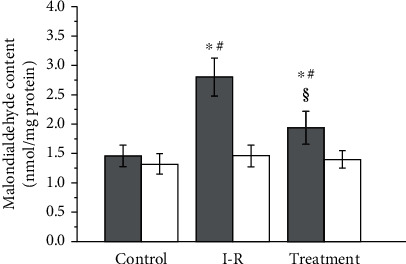
Malondialdehyde content in bilateral testes from rats in the sham-operated control, testicular ischemia-reperfusion (I-R), and salidroside-treated groups. Grey and white histograms represent ipsilateral and contralateral testes, respectively. Data (*n* = 10) are expressed as mean ± standard deviation. ^∗^Significantly different when compared with control group (*P* < 0.001). ^#^Significantly different when compared with contralateral testes in same group (*P* < 0.001). ^§^Significantly different when compared with ipsilateral testes in I-R group (*P* < 0.001).

Testicular malondialdehyde value in sham-operated control, testicular ischemia-reperfusion, and salidroside-treated groups is presented in Figure 1. Malondialdehyde value was significantly higher in ipsilateral torsional testes in testicular ischemia-reperfusion group than in sham-operated control group (*P* < 0.001). Malondialdehyde value of ipsilateral testes in the salidroside-treated group was significantly lower than that in the testicular ischemia-reperfusion group (*P* < 0.001). However, statistically significant difference was not seen among three groups in terms of malondialdehyde value of contralateral nontorsional testes (*P* = 0.1966).

### 3.2. Superoxide Dismutase and Catalase Protein Expression in Testis

**Figure 2:**
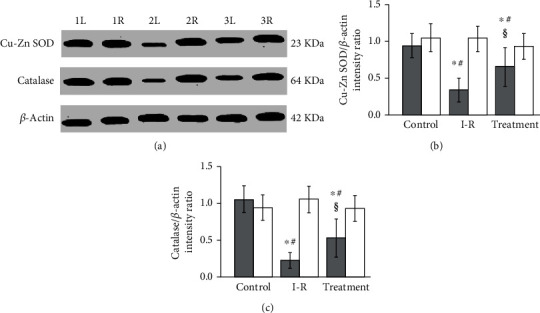
Western blot analysis for copper- and zinc-containing superoxide dismutase (Cu-Zn SOD) and catalase protein expression in testicular tissue. (a) Representative autoradiographs show Cu-Zn SOD and catalase protein expression in rat testes of sham-operated control, testicular ischemia-reperfusion (I-R), and salidroside-treated groups. The *β*-actin protein serves as a loading reference. Lanes 1L and 1R represent left (i.e., ipsilateral) and right (i.e., contralateral) testes in sham-operated control group. Lanes 2L and 2R represent ipsilateral and contralateral testes in testicular I-R group. Lanes 3L and 3R represent ipsilateral and contralateral testes in salidroside-treated group. Histograms of data display testicular Cu-Zn SOD (b) and catalase (c) protein expression in sham-operated control, testicular I-R, and salidroside-treated groups. The intensity ratio of Cu-Zn SOD or catalase band to *β*-actin band shows a relative expression level of Cu-Zn SOD or catalase protein. Grey and white histograms represent ipsilateral and contralateral testes, respectively. Data (*n* = 10) are expressed as mean ± standard deviation. ^∗^Significantly different when compared with control group (*P* < 0.01). ^#^Significantly different when compared with contralateral testes in same group (*P* < 0.05). ^§^Significantly different when compared with ipsilateral testes in I-R group (*P* < 0.01).

As shown in Figure 2, the expression in superoxide dismutase and catalase was significantly downregulated in ipsilateral torsional testes of testicular ischemia-reperfusion group compared with sham-operated control group (*P* < 0.001). In the salidroside-treated group, a significant increase was found in the superoxide dismutase and catalase expression of the ipsilateral testes as compared with that in the testicular ischemia-reperfusion group (*P* < 0.01). In contrast, statistically significant difference was not detected among three groups in terms of superoxide dismutase and catalase expression of contralateral nontorsional testes (*P* = 0.2829 and *P* = 0.2328, respectively).

### 3.3. Testicular Spermatogenesis

**Figure 3:**
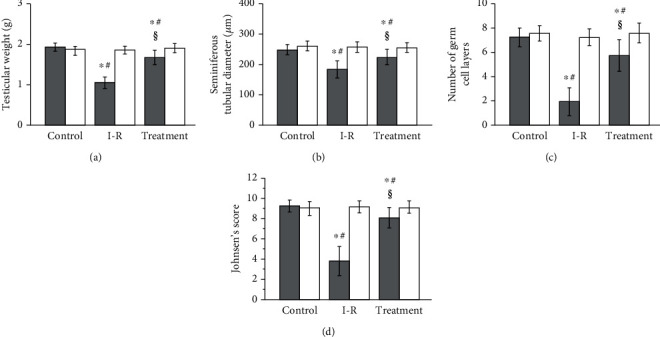
Testicular weight (a), seminiferous tubular diameter (b), number of germ cell layers (c), and Johnsen's score (d) in bilateral testes from rats in the sham-operated control, testicular ischemia-reperfusion (I-R), and salidroside-treated groups. Grey and white histograms represent ipsilateral and contralateral testes, respectively. Data (*n* = 10) are expressed as mean ± standard deviation. ^∗^Significantly different when compared with control group (*P* < 0.05). ^#^Significantly different when compared with contralateral testes in same group (*P* < 0.05). ^§^Significantly different when compared with ipsilateral testes in I-R group (*P* < 0.001).

**Figure 4:**
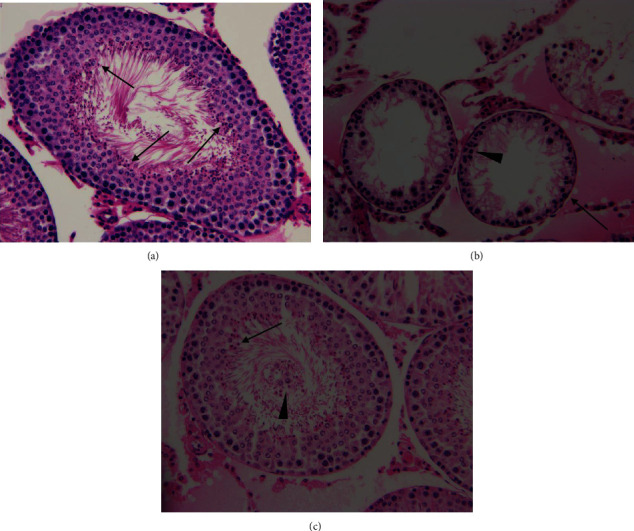
Representative photomicrographs of testicular histology in sham-operated control, testicular ischemia-reperfusion, and salidroside-treated groups. (a) In bilateral testes of sham-operated control group and contralateral testes of testicular ischemia-reperfusion and salidroside-treated groups, normal appearance in both seminiferous tubular diameter and germ cell layer number was observed. In seminiferous tubule, there were a lot of mature spermatozoa (arrows) and an open tubular lumen. (b) Ipsilateral testes in testicular ischemia-reperfusion group displayed notable seminiferous tubular atrophy (arrow) and a remarkable reduction in germ cell layer number (arrowhead). Spermatogenesis was interrupted at the early stage (arrowhead). (c) In ipsilateral testes of salidroside-treated group, seminiferous tubular architecture was almost up to normal standard. Mature spermatozoa (arrow) were seen in seminiferous tubule. However, seminiferous tubule contained a number of sloughed germinal cells (arrowhead) in tubular lumen, which caused seminiferous tubular obstruction easily. Testicular cross sections were stained with hematoxylin and eosin and observed under original magnification (×200).

As shown in Figures 3 and 4, the ipsilateral torsional testes from testicular ischemia-reperfusion group showed significantly lower testicular weight, seminiferous tubular diameter, germ cell layer number, and Johnsen's score compared with sham-operated control group (*P* < 0.001). The four parameters in the ipsilateral testes in the salidroside-treated group were significantly higher than those in the testicular ischemia-reperfusion group (*P* < 0.001). The four parameters in the contralateral nontorsional testes did not show any significant difference among three groups (*P* = 0.5375, *P* = 0.7895, *P* = 0.4644, and *P* = 0.8790, respectively).

## 4. Discussion

Testicular torsion interrupts blood supply to the testis and leads to testicular ischemia. Quick surgical detorsion is the main treatment approach of testicular torsion. If surgical detorsion is carried out within 6 hours after symptomatic onset, 90%-100% of testes will be saved [36]. However, testicular salvage rate falls to 20%-50% if treatment is performed within 6-12 hours [36]. Treatment within 12-24 hours can only gain testicular salvage rate of 0%-10% [36]. Testicular atrophy still occurs postoperatively in 13.5%-68% of such patients even if surgical detorsion is successfully carried out [4–8]. In our study, surgical detorsion was performed after 2 hours of testicular torsion. Despite testicular survival after torsion-detorsion, impaired spermatogenesis in ipsilateral testes was detected 3 months after detorsion. Impaired spermatogenesis was indicated by significant decreases in testicular weight, seminiferous tubular diameter, germ cell layer number, and Johnsen's score (Figures 3 and 4).

Testicular damage caused by torsion and detorsion is regarded as a classical ischemia-reperfusion injury. Testicular ischemia-reperfusion causes overproduction of reactive oxygen species [9–12]. Excessive reactive oxygen species have a destructive effect on cells by inducing peroxidation of cellular membrane lipids, DNA disintegration, and protein denaturation [13]. Reactive oxygen species are short-lived oxidizing agents because of their high reactivity and high instability [37]. Therefore, it is extremely difficult to quantify reactive oxygen species directly. Malondialdehyde, a stable byproduct of cell membrane lipid peroxidation produced by reactive oxygen species, has been extensively accepted as a reliable index of reactive oxygen species [38–41]. Our study found that rats in testicular ischemia-reperfusion group showed significantly higher malondialdehyde level and lower spermatogenesis in ipsilateral testes, as compared with sham-operated control group (Figures 1, 3, and 4). These data strongly suggest that injury of testicular spermatogenesis after testicular ischemia-reperfusion is due to overproduction of reactive oxygen species. Previous studies have demonstrated that treatment with reactive oxygen species scavengers can reduce ischemia-reperfusion injury in the liver, kidney, heart, brain, and so on [42–45].

Salidroside, a potent antioxidant, has been demonstrated to lessen ischemia-reperfusion injury in the heart, brain, liver, and spinal cord [27–30]. For this reason, we tried to explore the effect of salidroside treatment on testicular ischemia-reperfusion injury in a rat testicular torsion-detorsion model. Our study found that rats treated with salidroside had significantly reduced malondialdehyde level and had significantly increased spermatogenesis in the ipsilateral testes, as compared with rats in testicular ischemia-reperfusion group (Figures 1, 3, and 4). These results reveal that salidroside protects testicular tissue from ischemia-reperfusion injury through decreasing reactive oxygen species level. Epirubicin is an effective chemotherapeutic drug for the treatment of breast cancer [46]. Researchers have found that salidroside can protect patients with breast cancer against epirubicin-induced early left ventricular regional systolic dysfunction by its antioxidative activity [46]. No clinical adverse events were observed during salidroside therapy [46]. Taken together, these results indicate that salidroside may be a promising candidate for treating testicular ischemia-reperfusion injury in clinical practice. Nevertheless, the mechanisms by which salidroside reduces reactive oxygen species level have not been fully understood.

Reactive oxygen species are produced in most aerobic organisms [47]. The biological functions of reactive oxygen species depend on their concentration [48]. Under normal conditions, reactive oxygen species at low concentration are indispensable for physiological processes, such as killing bacteria, cell differentiation, activating transcription factors in signal transduction pathways, cell proliferation, apoptosis, and protein phosphorylation [49–56]. However, under pathological conditions, such as tissular ischemia-reperfusion, excessive reactive oxygen species are produced [9–12]. Reactive oxygen species at high concentration can destroy cellular macromolecules, including lipids, proteins, and nucleic acids, and lead to cellular damage [13]. To protect against reactive oxygen species-mediated injury, aerobic organisms have developed an effective antioxidant defence system [57–60]. Both superoxide dismutase and catalase are primary antioxidant enzymes in the antioxidant defence system [57–60]. Superoxide dismutase catalyzes dismutation of superoxide anion into hydrogen peroxide and oxygen [61, 62]. Then, catalase catalyzes the decomposition of hydrogen peroxide into oxygen and water [63]. If catalase is absent in aerobic organisms, hydrogen peroxide will be converted to highly toxic hydroxyl radical by the Fenton reaction in the presence of Fe^2+^ [64]. Thus, superoxide dismutase and catalase work together to scavenge reactive oxygen species and maintain a balance between reactive oxygen species generation and their elimination, thereby effectively protecting cells against oxidative damage [63]. When overproduction of reactive oxygen species overwhelms the defence ability of antioxidant system, the oxidative stress occurs and leads to tissular damage [65, 66]. Our study showed that significantly higher malondialdehyde concentration and significantly lower superoxide dismutase and catalase protein expression in ipsilateral testes were observed in testicular ischemia-reperfusion group, compared with sham-operated control group (Figures 1 and 2). These findings suggest that overproduced reactive oxygen species during testicular ischemia-reperfusion deplete these antioxidant enzymes. Our findings are in accord with the results of previous studies [67–70]. In addition, we found that superoxide dismutase and catalase protein expression significantly increased, while malondialdehyde concentration significantly decreased in ipsilateral testes of salidroside-treated group, compared with testicular ischemia-reperfusion group (Figures 1 and 2). These data indicate that salidroside decreases reactive oxygen species levels via upregulating the protein expression of superoxide dismutase and catalase.

Some studies have proved that salidroside at the dose of 20 mg/kg is effective in treating ischemia-reperfusion injury in rat heart, brain, and liver [27–29]. As a result, this dose was chosen in our rat model. In the present study, treatment with salidroside (20 mg/kg) provided partial rescue of ipsilateral testicular spermatogenesis, though this rescue was not complete (Figures 3 and 4). We did not evaluate the effect of salidroside on testicular ischemia-reperfusion injury at different doses and different administration times. Hence, further research is needed to investigate the optimal dose and administration times so that salidroside can achieve the best therapeutic effect.

Debate continues regarding whether contralateral testicular damage occurs after unilateral testicular ischemia-reperfusion. Some studies reported that contralateral testis was affected by unilateral testicular ischemia-reperfusion [71–74], but other studies showed no changes in the contralateral testis [75–77]. In our study, although unilateral testicular ischemia-reperfusion led to significant changes in malondialdehyde content, superoxide dismutase and catalase protein expression, and spermatogenesis in ipsilateral testis, it had no effect on contralateral testis (Figures 1–4). Consequently, we believe that unilateral testicular ischemia-reperfusion does not induce contralateral testicular injury.

*Rhodiola rosea* contains approximately 140 constituents, including salidroside, tyrosol, rosavin, gallic acid, and rosaline [16, 78]. Uyeturk et al. have reported that *Rhodiola rosea* extract has preventive effects on testicular ischemia-reperfusion injury [79]. In their study, *Rhodiola rosea* extract, Arctic root SHR-5 (containing salidroside, tyrosol, and rosavin), was used [79]. In our study, we examined the effect of salidroside (a major bioactive ingredient of *Rhodiola rosea*) on testicular ischemia-reperfusion injury. Our study showed that salidroside attenuated testicular ischemia-reperfusion injury (Figures 3 and 4). Whether the other ingredients of *Rhodiola rosea* have protective effect on testicular ischemia-reperfusion injury merits further investigation.

A control group receiving only salidroside treatment can help researchers to assess the basic effect of salidroside on normal testis. In our unilateral testicular ischemia-reperfusion + salidroside-treated group, we found that salidroside could reduce ipsilateral testicular ischemia-reperfusion injury (Figures 3 and 4). Nevertheless, salidroside had no any effect on contralateral normal testis (Figures 3 and 4). Hence, the control group receiving only salidroside treatment may be omitted in our study.

It has been reported that 2-hour 720° testicular torsion in a rat model can disrupt spermatogenesis [80]. Thus, we chose testicular torsion of 720° for 2 hours in our rat experiment. In addition, many other investigators studying testicular torsion-detorsion also chose the same duration of torsion and degree of torsion in the rat model as we did [81–85].

In our study, testicular spermatogenesis was assessed by some indicators, such as testicular weight, seminiferous tubular diameter, germ cell layer number, and Johnsen's testicular biopsy score. These indicators well showed testicular ischemia-reperfusion injury (Figures 3 and 4). They have been widely used in testicular ischemia-reperfusion injury studies [86–88]. Testicular section stained with Masson trichrome is usually used to quantify collagen accumulation as a marker of fibrosis [89]. Recent studies have shown that testicular ischemia-reperfusion can induce testicular fibrosis [89–92]. Therefore, testicular fibrosis is also a good indicator which shows testicular injury. We will try to use it in future study.

## 5. Conclusion

This is the first study to show that salidroside alleviates testicular ischemia-reperfusion injury in rats by upregulating superoxide dismutase and catalase expression to reduce reactive oxygen species content. Therefore, we propose that salidroside may have therapeutic application in patients suffering from testicular ischemia-reperfusion injury. However, additional clinical trials are needed to assess its efficacy for clinical use.

## Data Availability

All data used to support the findings of this study are included in the article.
